# The Book World of Medicine and Science

**Published:** 1897-05-29

**Authors:** 


### THE BOOK WORLD OF MEDICINE AND SCIENCE.

THE CO-EDUCATION OF THE SEXES. By MABEL HAWTREY. (London: Kegan Paul, Trench, Trubner, and Co , 1896.)

The object of this book is to show that co-education is not suitable for boys and girls when they are maturing, and the argument hinges largely upon this fact, that such training does not recognise the sex characteristics, and that in it the differences of their respective powers and their subsequent development are not kept in view. A considerable movement seems now to be in progress, having for its object the founding in this country of mixed schools for boys and girls, and this plan has been favourably commented upon by the Royal Commissioners who have lately been considering questions of secondary education. The question, however, is one which Miss Hawtrey thinks should not be confined to educationalists alone, but should be brought before the general public, on whom the decision must ultimately rest. Hence, we presume, this book. Education, we are told, and told very rightly, cannot be confined to the intellect merely, but must include the training of the physical and moral powers. But co-education must mean that boys and girls are to be taught the same things at the same time, in the same place, with the same methods, and under the same regimen, and although there may be no reason why such a method should not be adopted in the education of young children, or of young men and women, it is held that a great difficulty arises in making it suitable for boys and girls during the years when they are developing, since such training does not recognise sex and does not allow for differences in their respective organisations. This idea of the different direction in which development takes place in the two sexes, and as a consequence of the different training which is required at the time when such development is taking place, runs throughout the book, and there no doubt is a great deal in it. At the same time we do not feel that we can quite accept all the views put forward, nor can we believe that any real evil would result even if the daily association of boys and girls in the class-room should have the effect "of making the sexes regard one another with a feeling of indifference," however contrary to nature such a condition of mind may be. We feel quite sure that such a feeling of indifference will not last long. The book, however, is well worth reading, although to our mind the most interesting part of it lies in the criticisms of the boys and girls' schools of the present day, all of which, by the bye, tend rather to play into the hands of those who think that, at any rate, some change is desirable in current methods of education.

A PLEA FOR THE UNBORN. By HENRY SMITH. (London: Watts and Co. 1897. Pp. 102.)

This is a curious but not uninteresting book, obviously well-intentioned, but essentially unpractical. It opens with a simile from the medical profession which is now, as the author states, devoting its energies more and more to the prevention of disease with a view to lessening the amount requiring cure. Why, asks Mr. Smith, should not the same method be applied to crime? He regards all crime as the result of disease or imperfect development of the brain, and holds that the aim of legislators should be to produce a perfect man, one in whom the mental and physical faculties shall not only reach the acme of development but shall also be exactly balanced—a condition irresistibly suggesting a human "one-horse shay." This is to be effected by legislation prohibiting anyone who is mentally or physically diseased from marrying, or, to use the correction adopted by the author in a later pamphlet, from mating. The perfect man having thus been produced by State-aided selection and stirpiculture, crime and disease will alike vanish. To use the author's own words (p. 81), "We want a perfect man, and if we desire that perfect man we must breed him, and use the same means as the horse-breeder, the florist, and the pigeon-fancier." Critics have very pertinently pointed out that legislation such as Mr. Smith desires would be a very difficult matter. For such objections he has a lordly disdain; to remove the difficulties is none of his business; it is for the legislators to consider and dispose of them. It will thus be seen that practical capacity is not one of Mr. Smith's strong points; we fear that many of his theories also rest on perilously frail foundations. Thus he assumes that unhealthy people can only produce unhealthy offspring and that healthy parents cannot. Neither of these propositions is correct, for, to take only one example, cases are known of deaf mutes marrying and having normal children, while everyone knows of deformed and congenitally diseased children (*e.g.*, those suffering from hare-lip or congenital heart disease) born of apparently healthy progenitors. To remedy the latter evil the author proposes the destruction of unhealthy babies, but this is of no avail in the large number of cases in which an hereditary taint manifests itself only at puberty, or even later in life. Would it not be far more satisfactory to kill off all diseased or deformed individuals of whatever age at once? It would certainly from the author's point of view be more rational. The cardinal fault of the whole book is superficiality; it is obviously, like Mr. Benjamin Kidd's almost forgotten work on Social Evolution, written by a man whose knowledge of science, biological at any rate, is entirely derived from magazine articles. His final appeal for the rational basis of his theories is to—phrenology. Thus he says (pp. 58-9), "When the organs of the brain ['and heaven it knoweth what that may mean '] are their normal size man is perfect. To attain this state of perfection each organ must be fully developed. If one faculty is in excess, or deficient, the balance of the mind is lost." Hence follows legislative selection to develop the right organs and subdue the wrong ones. Small wonder that a man who holds such views should defy anyone to prove that any advance has been made in art or science during the last 6,000 years. We would gladly hail the day when some check should be placed upon the propagation of vice and disease, but we sadly fear that reform will be long delayed if it has many more such dangerous champions as Mr. Henry Smith.

TOXICOLOGY. Catechism Series. (Edinburgh: E. S. Livingstone. Price 1s. 48 pages.)

Owing to the greater importance that this branch of medicine is acquiring in the various medical examinations, the publication of this number of the Catechism series will be received with gratitude by intending candidates. It has been compiled on strictly practical lines, and being short and to the point can be read through in a few hours without any great degree of mental strain. The chemical side of the question has been, perhaps, too superficially dealt with, the formula and description of both organic and inorganic poisons being either omitted or briefly mentioned, but, as we said, it is the practical points of importance which are emphasised and made of primary importance.

